# Within- and Between-Individual Compliance in Mobile Health: Joint Modeling Approach to Nonrandom Missingness in an Intensive Longitudinal Observational Study

**DOI:** 10.2196/65350

**Published:** 2025-10-30

**Authors:** Young Won Cho, Sy-Miin Chow, Jixin Li, Wei-Lin Wang, Shirlene Wang, Linying Ji, Vernon M Chinchilli, Stephen S Intille, Genevieve Fridlund Dunton

**Affiliations:** 1 Department of Human Development and Family Studies The Pennsylvania State University University Park, PA United States; 2 Khoury College of Computer Sciences Northeastern University Boston, MA United States; 3 Department of Preventive Medicine University of Southern California Los Angeles, CA United States; 4 The Feinberg School of Medicine Northwestern University Chicago, IL United States; 5 Department of Psychology Montana State University Bozeman, MT United States; 6 Department of Public Health Sciences The Pennsylvania State University Hershey, PA United States; 7 Department of Population and Public Health Sciences University of Southern California Los Angeles, CA United States

**Keywords:** missing data, joint model, intensive longitudinal data, longitudinal data, mHealth, mobile health, smartwatch, digital health, digital intervention

## Abstract

**Background:**

Missing data are inevitable in mobile health (mHealth) and ubiquitous health (uHealth) research and are often driven by distinct within- and between-person factors that influence compliance. Understanding these distinct mechanisms underlying nonresponse can inform strategies to improve compliance and strengthen the validity of inferences about health behaviors. However, current missing data handling techniques rarely disentangle these different sources of nonresponse, especially when data are missing not at random.

**Objective:**

We demonstrate the usability of joint modeling in the mHealth context, showing how simultaneously accounting for the dynamics of health behavior and both within- and between-person missingness mechanisms can affect the validity of health behavior inferences. We also illustrate how joint modeling can inform distinct sources of (possibly nonignorable) missingness in studies using ecological momentary assessment and wearable devices. We provide a practical workflow for applying joint models to empirical data.

**Methods:**

We applied joint modeling on empirical data comprising 1 year of daily smartphone-based ecological momentary assessment data (affect and energetic feeling) and smartwatch-tracked physical activity (PA). The approach combined (1) a multilevel vector autoregressive model for examining the reciprocal influences between daily affect and PA, and (2) a multilevel probit model for missingness. Unlike conventional 2-stage imputation methods—which first impute missing data before fitting the main model—joint modeling handles missingness during model fitting without explicit imputation. Sensitivity analyses compared results from the proposed method to other missing data approaches that do not explicitly model missingness. A simulation study designed to mirror the temporally clustered (eg, consecutive days of missing data) and person-specific missingness patterns of the empirical data validated the feasibility of the proposed approach.

**Results:**

Sensitivity analysis indicated relative robustness of the autoregressive effects across missing data handling approaches, whereas cross-regressive effects could be detected only under the joint modeling but not with methods that did not simultaneously model missingness mechanisms. Specifically, under joint modeling approaches, participants had higher levels of PA on days following a previous day with higher self-report energy levels (95% credible interval [CrI] 0.012-0.049). Furthermore, the missing data model revealed both missing not at random and missing at random mechanisms. For example, lower PA predicted higher missingness in PA at the within-person level (95% CrI –1.528 to –1.441). Being employed was associated with higher missingness in device-tracked PA at the between-person level (95% CrI 0.148-0.574). Finally, simulation showed that joint modeling could improve the accuracy of estimates and identify nonignorable missingness.

**Conclusions:**

We recommend joint modeling with multilevel decomposition for addressing nonignorable missingness in mHealth/uHealth studies collecting intensive longitudinal data. We also suggest using a missing data model to explore the missingness mechanism and inform data collection strategies.

## Introduction

### Background

As technology advances, mobile health (mHealth) and ubiquitous health (uHealth) data collection via mobile and wearable devices has become increasingly common. This typically involves continuous tracking of physiological data, such as step counts and heart rate, through smartwatches [1], alongside self-reported ecological momentary assessment (EMA) surveys prompted on smartphones or web interfaces multiple times a day to measure self-reported behavioral and psychological variables [2,3]. Such intensive longitudinal data (ILD) [4,5] offer numerous advantages. They facilitate remote monitoring for timely interventions, reduce recall bias by capturing real-time data, and allow for examination of behavioral changes on more granular timescales. These features have the potential to enhance the effectiveness of health monitoring and research. Missing data are inevitable in mHealth and uHealth studies as triggered by myriad reasons, including variations in participants’ compliance, engagement, contextual factors, and other measurements and technical issues [6,7]. Whereas various modeling methods have been proposed to deal with missing data in ILD, few mHealth or uHealth studies have systematically leveraged and integrated passive device and subjective EMA responses to inform missingness mechanisms that drive across-participant and across-time variations in compliance rates and possible consequences on health behavior inference [7-10].

### Why Joint Modeling for mHealth and uHealth Studies?

#### Overview

**Figure 1:**
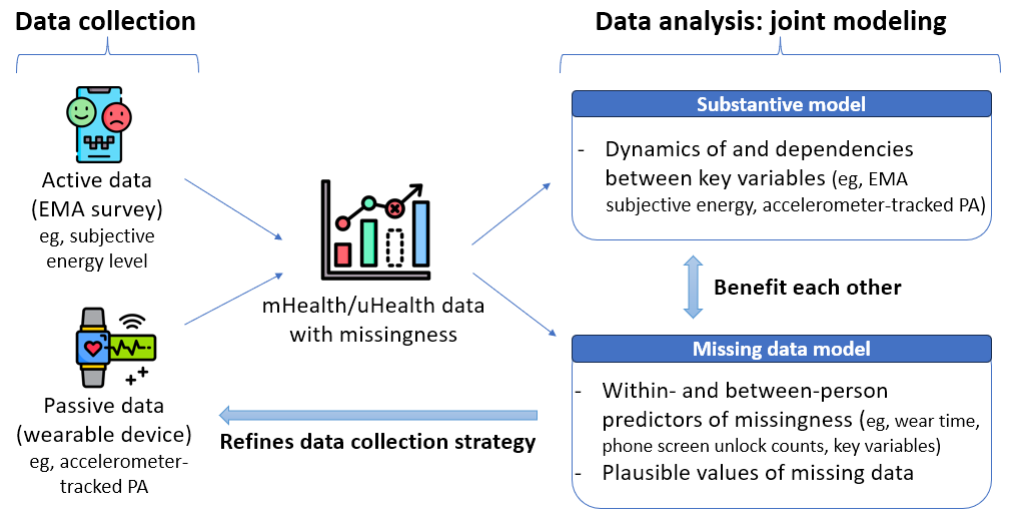
Conceptual framework of joint modeling. EMA: ecological momentary assessment; mHealth: mobile health; PA: physical activity; uHealth: ubiquitous health.

Joint modeling is a technique that handles nonresponse bias by simultaneously fitting 2 interconnected models: 1 for the main analysis (substantive model) and 1 for understanding how missing data might occur (missing data model). The substantive model could be any statistical model for testing the primary research question, such as linear regression, survival analysis, and path analysis. The missing data model is a model that examines whether missingness is linked to certain factors by modeling binary missing indicators of the variables of interest (see Figure 1 for an overview).

Joint modeling is commonly used to address nonignorable, also known as nonrandom missingness, which occurs when the missingness depends on unobserved outcomes. For example, if some participants wear their smartwatches only when they are physically active, the device-tracked physical activity (PA) data are said to be missing not at random (MNAR), as the likelihood of missingness depends on the unobserved PA levels, which ought to be measured but were missing on the less active days (alternatively, more germane scenarios include missingness completely at random [MCAR], in which the missingness does not depend on any variable, and missingness at random [MAR], in which the missingness depends only on variables that are observed in the measured data). Overlooking such nonignorable missingness by examining the substantive processes only with the available data often results in incorrect inferential conclusions [11].

Past mHealth and uHealth studies that sought to identify predictors of participant compliance [8,12] have typically focused exclusively on the “missing data models.” As such, the researchers miss opportunities to allow the substantive and missing data models to iteratively inform each other during model fitting, especially in MNAR scenarios.

Such a joint inferential approach has several advantages. First, by explicitly accounting for when and why missingness occurs, the missing data model informs substantive models with more plausible values for unobserved data, leading to enhanced estimates of key substantive processes (such as PA and energy levels), even when data are incomplete. Second, the missing data model also benefits from the substantive model, especially when the likelihood of missingness depends on variations in the substantive processes (ie, MNAR), as noted in several EMA studies involving subjective self-reports [13,14].

Third, in mHealth or uHealth contexts, this mutual reinforcement between models can be further enhanced by leveraging passively collected data. Passive data such as device usage indicators (eg, app engagement records) can be collected unobtrusively and may provide valuable auxiliary information for modeling missingness mechanisms.

Fourth, the enhanced ability to detect nonignorable missingness can inform better data collection strategies. For instance, if the model reveals that PA data are more likely to be missing during low-activity days (ie, less inclined to wear devices on inactive days; MNAR), researchers might implement targeted strategies to improve data quality, such as sending adaptive reminders encouraging device use particularly during low-activity periods. While direct follow-up with nonrespondents is ideal [15], it is often costly or infeasible, making joint modeling a valuable alternative for identifying potential MNAR without extra data collection.

Finally, even highly engaged participants exhibit occasional missingness in longitudinal studies, but no studies to date have considered the implications of conflating within- and between-participant variations in compliance. Examining how compliance fluctuations relate to PA and energy levels can inform the design of more sustainable health behavior interventions.

This study capitalizes on a 1-year intensive longitudinal study to demonstrate how device-tracked PA and subjective EMA reports (eg, energy levels) can be integrated within a joint modeling framework that captures both within- and between-person variations. Passive indicators such as cell phone unlock counts are incorporated as auxiliary variables to aid modeling missingness. This approach highlights the benefits of leveraging multiple data sources and the risks of ignoring key sources of variation.

#### Existing Joint Modeling Approaches

Three common approaches for handling missing data within the joint modeling framework are the selection model, shared parameter model (SPM), and pattern mixture model (PMM). We focus on the selection model and SPM, as PMM is less applicable to ILD context. PMM stratifies data by missing patterns and forms distinct models for each [16], which becomes impractical for ILD, given its diverse missing patterns.

In the selection model, we assume that the missingness depends on the unobserved values of the dependent variables (DVs). Thus, the selection model directly models the probability of missingness as a function of the (possibly unobserved) DV and uses a joint likelihood to link the substantive and missing data models [17]. For example, participants may be inclined to wear their smartwatches tracking PA only when they are physically active, leading to missing PA data when they are sedentary. To address this MNAR issue, selection models directly incorporate PA levels as a predictor in the PA missingness model, thereby correcting the bias caused by nonignorable missingness. Additionally, by integrating auxiliary variables, additional variables not part of the main analysis but providing valuable information about the missing data mechanism, as covariates [18] (eg, time of day as a proxy for habitual exercise routines), the model can also capture and adjust for MAR mechanisms. This approach enables the missing data model to serve as a tool not only for correcting bias but also for investigating potential contributors to the missingness, such as how PA levels influence the likelihood of PA data being missing.

In SPM, the missingness and substantive models are linked through shared latent parameters (eg, random effects) [19], and it assumes that the substantive and missingness processes are independent, given the shared or correlated random effects. For instance, in a longitudinal treatment study, patients may be more likely to drop out as their health deteriorates (MNAR). Here, the patient’s latent health trajectory—represented by a random slope (ie, person-specific effects of time on health)—is associated with data missingness. By incorporating an individual’s random slope as a predictor in the missing data model, 2 processes are linked [20]. When the missing data model’s regression coefficient for the random slope is nonzero, it indicates dependence between the substantive and missingness processes.

While SPM and the selection model are often described as distinct approaches, SPM is sometimes viewed as a variant of the selection model [21,22]. Specifically, when applying a selection model within a multilevel framework, the structure closely resembles that of the SPM [22]. This similarity arises when the DV is decomposed into within- and between-person components, and the between-person component (ie, the random intercept) is used as a predictor in the missing data model. In this case, it mirrors SPM’s use of a latent random effect to link missingness and substantive processes.

These approaches form the conceptual basis of our proposed joint modeling, which specifically targets within- and between-person heterogeneity, as well as temporally clustered missingness—the tendency for missing days to be followed by more missing days. We describe each of these characteristics in turn.

### Missing Data Issues in ILD

#### Heterogeneity in Missing Data Patterns Within and Across Individuals

Unlike conventional panel longitudinal data, ILD is characterized by a much higher frequency of assessments, leading to greater variability in missing data patterns. First, a high frequency of data collection can amplify within-person variations in compliance because the number of possible missing data patterns increases with an increase in the number of assessment occasions. Since data are collected daily or multiple days a time, more factors can influence daily compliance rates in mHealth and uHealth studies [8,9], such as daily variations in affect, technical issues, forgetting to wear a watch [23], or any disruptions in daily lives (eg, illness, vacations). Nahum-Shani et al [7] discussed how engagement in digital interventions using mobile devices can be influenced by contextual factors such as emotional, physical, and cognitive energy. As more concrete examples, nonresponse to EMA push notifications could be influenced by the emotional energy of the survey moment, as well as PA levels or sleep duration on the day, which is proxy of physical energy throughout the day.

Second, participants may miss assessment points due to baseline individual differences in compliance, such as levels of conscientiousness or familiarity with mobile technology. This between-person variation can become more pronounced with more repeated measures. Martinez et al [12] reported that compliance rates for wearable trackers and mobile EMA were related to person-specific characteristics. Older participants tended to have more difficulties using smartphones, leading to lower compliance rates [24]. A higher BMI was related to lower compliance rates in a weight intervention study because of physical discomfort with wearing the device and the collection of weight data. In summary, participants’ compliance to mHealth and uHealth data collection changes not only because of their person-level traits but also due to changes in daily contextual factors. As sample size and measurement occasions increase, so does the variability in compliance rates both within and across individuals.

Our proposed joint modeling approach allows for between- and within-person heterogeneity in missingness by combining a multilevel missing data model with a multilevel vector autoregressive (VAR) model for the substantive processes of interest. Applications of such multilevel decomposition of missingness are still nascent in the literature and are primarily restricted to cross-sectional settings [25], or iterations through missing data imputation and substantive process modeling in 2 stages [23]. Although a few studies in the ILD literature have adopted joint modeling approaches (with substantive process models that were similar to that adopted here) [26], these studies did not apply multilevel decomposition to the missing data model itself.

#### Temporally Clustered Missingness

**Figure 2:**
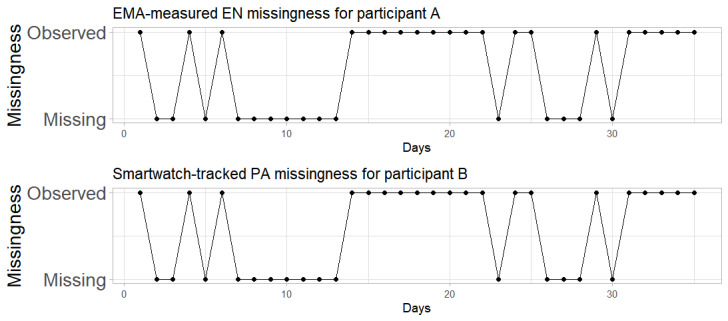
Data missingness in bursts from 2 participants. EMA: ecological momentary assessment; EN: energetic feeling; PA: physical activity.

In our empirical data, we observed significant patterns of temporally clustered missingness (see Figure 2 for examples). Specifically, 25.2% (55/218) of the participants had at least 1 period of 7 consecutive days of missingness for EMA-measured energetic feelings (ENs), and 41.3% (90/218) of participants had at least 1 period of 7 consecutive days of missingness for smartwatch-tracked accelerometer-measured PA (see section “Motivating Empirical Data” for the valid data criterion). These patterns highlight a notable feature of missing data in ILD: burst or temporally clustered missingness, where 1 day of missingness tends to be followed by more missing days. This clustering can occur for various reasons. For instance, during busy periods or while traveling, participants may ignore survey prompts for several consecutive days. If any technical issues occur, the data can be continuously missing until the problem is resolved. Such burst missingness suggests that each instance of missing data may not be independent of the previous instances.

Incorporating this pattern into missingness models could improve the accuracy of analyses. However, existing methods often overlook these patterns, likely because it is not commonly observed in cross-sectional or conventional panel longitudinal data. To our knowledge, only 1 study has considered the temporal dependency of missingness when modeling missing processes but without substantive models [27]. In contrast, an ILD study with a joint modeling approach [26] did not incorporate such a temporal component in its missing data model. To address this gap, we integrate autoregressive (AR) terms into our proposed missing data model within the framework of the joint model. AR terms account for the autocorrelation in missing data by relating the current missing data status to the previous time points, thereby capturing the tendency for missingness to occur in clusters.

Our approach is a multilevel joint model based on the selection model framework, assuming the missingness in DV depends on unobserved variables or parameters (MNAR), and other observed covariates (thus allowing for MAR as a special case). Under this approach, substantive and missingness models are connected via joint likelihood and correlated residuals. While rooted in the selection model framework, our multilevel joint model shares some similarities with SPM: DVs are decomposed into within- and between-person components in substantive models, and the latent between-person components (ie, random intercepts) serve as predictors in missing data models. Our missing data model can be further extended from an SPM perspective by incorporating additional random effects (eg, random AR effects in VAR models). However, such an extension was not implemented, due to the lack of theoretical background to expect that person-specific AR coefficients would be systemically related to missingness. Table 1 compares the selection model, SPM, and our multilevel joint model, emphasizing their suitability for different scenarios and highlighting the unique contributions of our approach.

**Table 1:** Comparison of joint modeling missing data handling approaches.

	Key properties	When to use	Common use cases
Selection model	Links outcome and missingness models via joint likelihood, often with correlated residuals.	When missingness depends on unobserved DVs^a^.	Widely applied in cross-sectional and panel data.
Shared parameter model	Models share latent parameters (eg, random effects). Assumes conditional independence given shared parameters.	When missingness relates to latent change components (eg, random slope).	Typically to model dropout or attrition in longitudinal panel studies. Terminal dropout or attrition does not require consideration of overtime variations in missingness.
Multilevel joint model (this study)	Link 2 models via joint likelihood and correlated residuals. Does not assume conditional independence given shared parameters. Decomposes missingness into within- and between-person levels, disentangling momentary and habitual missing tendencies.	When missingness depends on either unobserved variable values or latent changing dynamics (eg, random AR^b^ effects), with temporally clustered missing patterns.	Tailored for ILD^c^ with intermittent missingness. Useful for ILD-specific challenges: mixed sources of missingness (person- and context-level), temporal dependency of missingness.

^a^DVs: dependent variables.

^b^AR: autoregressive.

^c^ILD: intensive longitudinal data.

### This Study

Due to the distinct patterns of missingness in ILD, specialized approaches are required for handling missing data. In this study, we apply multilevel joint modeling to empirical mHealth data consisting of up to a year of daily self-reports on feeling energetic and PA, tracked by smartwatches. Especially, through this empirical analysis, we provide practical guidance on building missing data models and discuss the use of joint modeling of the dynamics of the substantive health processes and missingness mechanisms.

Additionally, by comparing the empirical results obtained across different missing data handling methods—such as listwise deletion, last observation carried forward (LOCF), mean imputation, Bayesian full information maximum likelihood (FIML), and joint modeling with alternative missingness models—we highlight the importance of conducting sensitivity analysis to evaluate the robustness of substantive conclusions.

Given that true substantive and missing data models are never known in empirical analysis, we further supplement the empirical analysis with a simulation study. This simulation aims to verify the efficacy of the joint modeling approach under known data-generating mechanisms and missing data patterns designed to mirror the intricacies of empirical ILD, such as individual variations in compliance rates, clustered patterns of missingness, and the presence of ordinal DV (eg, Likert scale).

## Methods

### Motivating Empirical Data

Data from the Temporal Influences on Movement and Exercise study [28], collected over 1 year, were analyzed. The study recruited young adults (aged 18-30 years; N=246) across the United States. Participants completed smartphone EMA surveys on EN at the end of the day and wore smartwatches to track PA throughout their participation. Each participant’s study start date was defined as the first day with both EMA and wearable data. If EMA responses continued after wearable data had permanently stopped (eg, due to technical issues), those trailing EMA entries were excluded to preserve meaningful variation in both affect and PA.

Data were retained for participants who had at least 14 days of nonmissing data for both EN and PA and at least 30 days of participation. Participants with more than 80% missing data in either EN or PA were considered noninformative for the intended analysis and excluded to prevent excessive imputation bias. This yielded final data from 218 out of 246 individuals, with data ranging from 39 to 401 (median 323.5, IQR 153.5-347) days and a total of 55,716 data points.

#### Measurement

Participants rated their daily EN on a 5-point Likert scale via smartphone app at the end of the day. Smartwatch-tracked PAs were first summarized using the motion independent movement summary units [29], based on raw acceleration data from Fossil Sport 4/5 (initially in 1-second epoch), and were aggregated daily. To facilitate efficient model fitting, daily PA was scaled by its standard deviation to align with other variables, as large discrepancies in variable scales can lead to numerical instability and slower convergence in complex models [30].

Time-varying covariates included daily device wear time, self-reported daily sleep duration (in hours), a season indicator, and phone screen unlock counts per day. Device wear time was determined from the SWaN (Sleep Wake and Nonwear) algorithm [28], which categorized available data into Wear, Sleep, or Nonwear. Demographic and between-person variables were also considered: sex (female=1 and male=0), age, employment status (employed for wages=1 and no=0), self-reported depression diagnosis (yes=1 and no=0), and alcohol consumption habit (number of days of drinks per week).

#### Missing Data

Individual missing data percentages for EN (the proportion of missing days relative to each participant’s total participation) ranged from 0% to 71.2%, with an average of 16.7% per person. For the entire dataset, the missing rate was 14.9% (8319/55,716), with continuous missing days ranging from 1 to 38 (mean 1.6, SD 1.98 days). For daily PA, missing data were defined as less than 10 hours of smartwatch wear [31]. If no data were available, it was treated as 0 wear time, and PA was marked as missing. Individual missing data percentages for PA ranged from 0% to 68.5% (average=19.5% per person). For the entire dataset, the missing rate was 18.4% (10,265/55,716), with continuous missing days ranging from 1 to 100 (mean 2.04, SD 3.56 days). The median of the continuous missing days was 6, and the 90th percentile was 30, indicating that most of the gaps were short, with only 10% exceeding a month. Notably large gaps (eg, 100), upon reviewing research notes, were confirmed to be due to technical issues, with participants resuming after the issues were resolved.

### Ethical Considerations

This study analyzed fully anonymized secondary data obtained from a previous study [28] conducted at the University of Southern California (HS-18-00605). The original study was approved by the University of Southern California Institutional Review Board and conducted in accordance with the ethical standards laid down in the 1964 Declaration of Helsinki and its later amendments. All participants provided informed consent to have their deidentified data published in journals. As this study involved only anonymized secondary data and did not include direct participant contact, additional ethical review was not required.

### Statistical Analysis

**Figure 3:**
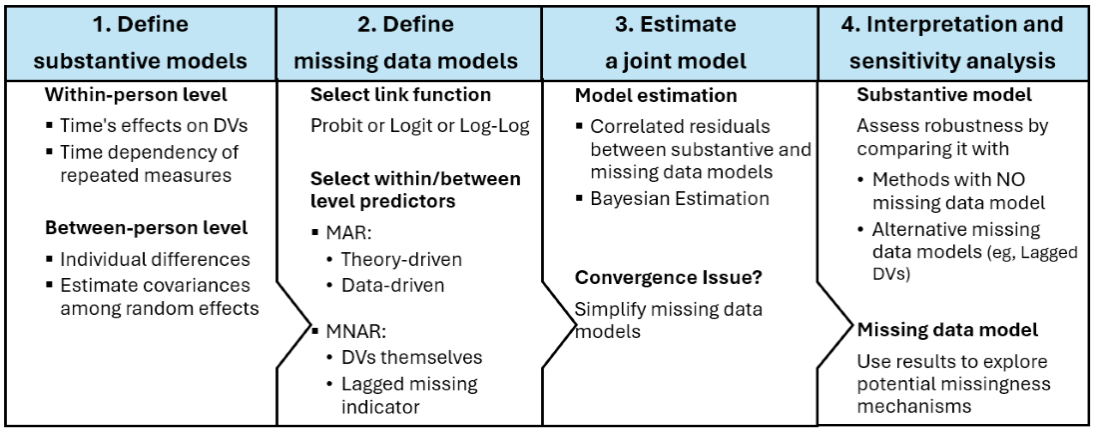
Workflow of multilevel joint modeling for handling missing not at random intermittent missingness. DVs: dependent variables; MAR: missing at random; MNAR: missing not at random.

Figure 3 provides an overview of our analytic framework. It outlines the key steps for specifying the substantive and missing data models, estimating them jointly, conducting sensitivity analysis, and interpreting the results. The following subsections elaborate on each step of this workflow. Code for replicating empirical analysis is available in Multimedia Appendix 1.

#### Substantive Model: Multilevel VAR (1) Model

Joint modeling involves 2 components: substantive and missing data models. The substantive model, driven by research questions, is a statistical model that tests research hypotheses. In our case, the research question focused on the reciprocal relationship between daily PA and EN over time. Specifically, we aimed to understand how the PA and EN from the previous day influenced those on the following day. This exploration of lead-lag relations can provide insights into how changes in one process may influence the other, potentially informing interventions to improve physical and mental well-being. For this, we used a VAR model as our substantive model. The VAR (1) model for EN (EN*_i_*_,_*_t_*) and PA (PA*_i_*_,_*_t_*) is described by the following equations. The subscripts *i* and *t* denote the values for *i*th individual at time *t.*



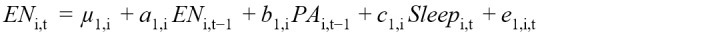





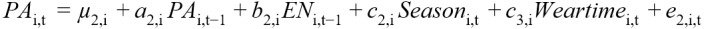



In these equations, *µ*_1,_*_i_* and *µ*_2,_*_i_* are individual baseline levels of EN and PA, when all predictors are at their average values. Individual variations in baseline were allowed and predicted by person-specific variables (sex, age, employment status, depression, and alcohol consumption habits), with EN baseline additionally predicted by person-average sleep duration, and PA baseline by person-average device wear time.

The coefficients *a*_1,_*_i_* and *a*_2,_*_i_* represent the AR effects for person *i,* namely, the extent of yesterday’s energy (PA) on today’s energy (PA), respectively. The coefficients, *b*_1,_*_i_* and *b*_2,_*_i_*, termed cross-regressive (CR) coefficients, represent yesterday’s PA → today’s energy and yesterday’s energy → today’s PA effects for person *i*. Thus, temporal dependencies within individuals were explicitly accounted for through individual differences in AR and CR effects, which include the sample’s average AR and CR coefficients (termed “fixed effects”), and stochastic parts that capture each person’s deviations from the sample averages (termed “random effects”). All the random effects were allowed to covary with each other.

Daily sleep duration, a seasonal indicator, and device wear time were included as time-varying predictors. The terms *e*_1,_*_i_*_,_*_t_* and *e*_2,_*_i_*_,_*_t_* represent dynamic noises, or residuals in the 2 processes that were not accounted for by other variables, but whose effects carried on to future time points through the AR and CR terms. These dynamic noises were allowed to covary with each other.

Given the importance of modeling time effects in ILD analyses, we examined potential temporal trends in PA. We found no significant linear trend in PA over the year. However, to account for seasonal variations in PA, we included a season variable in the model [32]. Exploratory analysis showed that PA levels were higher in summer and lower in winter, with no significant difference between spring and fall. Thus, winter was coded as –1, spring or fall as 0, and summer as 1. It is worth noting that the specification of the substantive model and the omission or misestimation of covariances between random effects can impact parameter estimates, particularly in the presence of substantial missing data [33].

#### Missing Data Model: Multilevel Probit Model

##### Choice of Models for Binary Outcomes

We constructed a missing data model for each of 2 DVs (EN and PA). Each was constructed as a multilevel univariate probit regression model [27], with the binary missing indicators of whether a data point is missing (0=observed and 1=missing) on a particular day *t* for person *i* and construct *d* serving as the ordinal DV. Underlying the ordered binary responses for each construct were a normally distributed, time- and person-varying latent variable that represented each individual’s underlying and continuous tendency missingness on a given day, with missingness occurring if this tendency exceeds an estimated threshold parameter.

While logit and log-log links are viable alternatives for modeling binary outcomes, we selected the probit model for both theoretical and practical reasons. First, although logit and probit models often yield similar results [34], theoretical considerations favor the probit model. Theoretically, the probit model aligns well with ILD missing data contexts, where a latent tendency for missingness at person level (habitual missing) and day level (short-term tendency) is plausible. Second, prior research suggests that in complex settings—such as multivariate binary response models with random effects—probit often provides better fit than logit [35]. Third, the log-log link is tailored to rare‐event or survival‐type dropout contexts, whereas our focus is on intermittent nonresponse. Finally, software constraints—such as Mplus’s support for multilevel Bayesian probit but not logit—made probit the most feasible option in practice.

##### Across-Time and Person Heterogeneity in Missingness

Since missing data indicators were themselves longitudinal and nested within each person, the proposed multilevel probit regression model extended standard probit regression by decomposing the missingness mechanisms into day- and person-level missingness. The within-person part modeled day-to-day variations in missing tendency related to time-varying covariates, such as energy and PA levels themselves, and phone usage. The between-person part captures individual differences in overall missingness tendencies, linked to person-specific predictors (eg, person mean of phone usage) and demographic factors. Table 2 summarizes representative hypotheses about within- and between-person missingness mechanisms and shows how each is reflected in the multilevel missing data model.

**Table 2:** Plausible missingness-related hypotheses.

Levels and hypotheses			Reflecting missing data models		Mechanisms
Within-person					
	On days when they feel less energetic than usual, they tend to miss more EMA^a^ prompts.	EN missing_*i,t*_ = β_1_ × EN^b^_*i,t*_		MNAR^c^	
	On days when they engage in more physical activity than usual, they tend to miss more EMA prompts.	EN missing_*i,t*_ = β_2_ × PA^d^_*i,t*_		MAR^e^, if PA missingness is not related to energy level. MNAR, if PA missingness is related to energy level itself (eg, on low-energy days, people are less likely to wear smartwatches).	
	On days when they use smartphones more than usual, they tend to miss fewer EMA prompts.	EN missing^f^_*i,t*_ = β_3_ × Phone^g^_*i,t*_		MAR	
Between-person					
	People who have lower energy than others tends to miss more EMA prompts.	EN missing^h^_*i*_ = β_5_ × EN_*i*_		MNAR	
	People who are generally more physically active than others tend to miss more EMA prompts.	EN missing_*i*_ = β_4_ × PA_*i*_		MAR, if overall PA missingness is not related to overall energy level. MNAR, if overall PA missingness is related to overall EN level (eg, those with generally lower energy are less likely to wear smartwatches).	
	People who generally use phones more than others tend to miss fewer EMA prompts.	EN missing_*i*_ = β_6_ × Phone_*i*_		MAR	

^a^EMA: ecological momentary assessment.

^b^EN*_i,t_*: energetic feelings. Subscripts *i* and *t* denote the values for *i*_th_ individual at time *t*.

^c^MNAR: missing not at random.

^d^PA*_i,t_*: physical activity. Subscripts *i* and *t* denote the values for *i*_th_ individual at time *t*.

^e^MAR: missing at random.

^f^Missing*_i,t_*: missing indicator at time *t* for person *i* (1=unobserved and 0=observed).

^g^Phone*_i,t_*: the number of times the phone screen was unlocked. Subscripts *i* and *t* denote the values for *i*_th_ individual at time *t*.

^h^Missing*_i_*: between-person level missingness for person *i*.

##### Selection of Predictors for Missing Data Models

Building missing data models requires identifying appropriate predictors of missingness, a process closely tied to hypothesis generation, as illustrated in Table 2. Theory-driven approaches use prior research to inform potential predictors of missingness or compliance. For example, age was related to lower compliance rates [24]. Daily emotional, physical, and cognitive energy may influence engagement in mHealth studies [7]. In our context, daily EN, PA, and sleep duration, which are proxies of daily energy levels, can be considered potential predictors of missingness. Also, smartphone usage had a significant impact on EMA response rates [8].

When theoretical guidance is limited, statistical exploration helps identify potential predictors of missingness, particularly under MAR assumptions. To inform the multilevel probit model, we conducted correlational analyses between missingness indicators and person-mean centered (within-person) and person-mean (between-person) variables. Predictors significantly associated with missingness at each level were selected for inclusion. The full list of predictors and their correlation results is reported in the “Results” section.

##### Burst Missingness

To capture burst patterns in missingness observed in the data, we included lagged (yesterday’s) latent missingness tendencies of yesterday’s missingness for EN and PA on today’s missingness for EN and PA in the missing data model. By incorporating temporal dependencies (ie, AR effects) of yesterday’s missingness for DVs on today’s missingness for DVs, the model allowed a participant’s missingness tendency on 1 day to influence missingness on the following day.

#### Estimation of the Joint Model

After constructing the substantive and missing data models, we jointly estimated them by linking them through joint likelihood and correlated residuals between the substantive and missing data models. For estimation, we used Bayesian methods due to its pragmatic advantages in handling complex models with multilevel structures [36-38]. Following practices for Bayesian reporting [39], we presented the median of each posterior distribution as the point estimate, alongside standard deviations and 95% credible intervals (CrIs). Parameters were considered significant if their CrI did not include zero.

Model estimation was conducted on a PC (Intel i7 CPU, 32GB RAM) and took 2 hours to converge. If there are too many predictors in the missing data model, the run time may increase and convergence issues may arise. In such cases, simplifying the model by reducing the number of predictors or restricting the random effects may help. In our experience, fitting the missing data model alone without the substantive model often led to convergence issues. This highlighted 1 practical advantage of using the joint modeling approach to allow inclusion of values of substantive processes in the missing data and vice versa to facilitate the estimation process. Details of the default Bayesian estimation settings in Mplus and the convergence criteria are outlined in Multimedia Appendix 1.

#### Sensitivity Analysis

To assess sensitivity to different missing data methods, we compared our joint modeling result with other commonly used techniques in behavioral science, including listwise deletion, LOCF, mean imputation, and Bayesian FIML. Listwise deletion (complete case analysis) removes any time points with missing values, resulting in the greatest extent of data loss among the methods considered. LOCF fills in missing values by carrying forward the last observed value, assuming no change since the last entry. Mean imputation replaces missing values with the variable’s overall mean, disregarding individual and temporal variation. FIML is a more sophisticated technique that leverages all available data, including both fully and partially observed patterns (where some variables are measured at a specific time point while others are missing). It is considered more robust than traditional methods, such as mean imputation, and effective under the MAR assumption [17]. Bayesian FIML is an approach that uses Bayesian inference to handle missing data similarly to FIML, leveraging the computational advantages of Bayesian estimation [40,41].

Additionally, given recent advancements in deep learning for time series imputation—particularly methods that explicitly model temporal dependencies—we conducted a sensitivity analysis using 2 methods that have shown promising results: SAITS (Self-Attention-based Imputation for Time Series [42]) and BRITS (Bidirectional Recurrent Imputation for Time Series [43]). SAITS is a transformer-based model that leverages self-attention mechanisms, while BRITS is based on bidirectional recurrent neural networks. We applied SAITS-base [42] and BRITS to impute missing values in our data before estimating the substantive model. Imputation was implemented using PyPOTS [44].

Notably, the methods discussed do not incorporate explicit missing data models that we can interpret. To further assess the sensitivity of our joint modeling approach, we examined the robustness of key conclusions under an alternative missing data model specification by extending the proposed missing data model to include additional lagged variables of the DVs as predictors.

Among the approaches mentioned, listwise deletion, LOCF, mean imputation, SAITS, and BRITS are 2-stage processes, where missing data are imputed first, followed by fitting the substantive model using the complete dataset. In contrast, Bayesian FIML and joint modeling are a 1-stage process that handles missing data while fitting the substantive model, without explicit data imputation process.

## Results

In this section, we first present the results from our proposed joint modeling approach, which addresses the possibility of data being MNAR, through both the substantive model and the missing data model. We then report the results of sensitivity analyses, comparing our approach with alternative missing data handling techniques and evaluating the robustness of our findings under different specifications of the missing data model within the joint modeling framework.

### Correlation Analysis for Specifying the Missing Data Model

To inform the multilevel probit model, we conducted correlational analyses between missingness indicators and both person-mean centered (within-person) and person-mean (between-person) variables. At the within-person level, daily phone usage (*r*=–0.065; *P*<.001) and sleep durations (*r*=0.021; *P*<.001) were correlated with EN missingness. Similarly, daily phone usage (*r*=–0.022; *P*<.001) and sleep durations (*r*=0.031; *P*<.001) were correlated with PA missingness. At the between-person level, average phone usage was negatively correlated with both EN (*r*=–0.255; *P*<.001) and PA (*r*=–0.14; *P*=.039). Average sleep duration was related to EN missingness (*r*=–0.136; *P*=.045). It should be noted that these correlational results are based on observed data only and may differ from results obtained under joint modeling; their purpose was to inform predictor selection rather than to test missingness mechanisms.

Based on the theoretical considerations and these correlational findings the final missing data models included the following predictors: at the within-person level, daily EN, PA, sleep, and phone usage, and at the between-person level, the person means of EN, PA, sleep, and phone usage, along with participants’ sex, age, employment status, self-reported depression, and alcohol consumption habit.

### Substantive Model Results

To examine the daily reciprocal relationship between PA and EN, the multilevel VAR (1) model was fitted jointly with the multilevel probit regression model to represent the missingness of 2 DVs. Results showed that higher than usual levels of PA or EN on 1 day were significantly associated with higher levels of the same variable on the following day, as indicated by positive AR coefficients (EN 0.226, 95% CrI 0.196-0.254; PA 0.081, 95% CrI 0.066-0.097). This suggests that both PA and EN exhibited a certain level of continuity over time. Regarding reciprocal influence—the focus of this analysis—CR coefficients indicated that higher energy levels on 1 day predicted higher PA levels the next day (*b*=0.03; 95% CrI 0.012-0.049), and vice versa: higher PA levels also predicted higher energy levels on the following day (*b*=0.03; 95% CrI 0.006-0.054).

Covariate effects were as follows. PA levels tended to increase during warmer seasons (*b*=0.031; 95% CrI 0.025-0.038). Individuals with self-reported depression exhibited generally lower EN levels (*b*=–0.411; 95% CrI–0.735 to –0.078). Individuals who were employed (*b*=0.380; 95% CrI 0.178-0.582) and those who reported higher frequencies of alcohol consumption (*b*=0.069; 95% CrI 0.009-0.128) showed higher overall PA levels. Additionally, sex differences were observed in PA baseline, with females exhibiting higher baseline PA than males (*b*=0.219; 95% CrI 0.017-0.420).

### Missing Data Model Results

In this section, we present results from missing data models to explore plausible hypotheses about missingness mechanisms. Full probit model results are shown in Table S1 in Multimedia Appendix 2. It is worth noting that the estimated missingness mechanisms here are not definitive; rather, they provide preliminary insights that warrant further discussion with experts in the field.

#### Missing Not at Random

The results indicated that the PA level was significantly associated with missingness in both within- and between-person levels of EN and PA. Lower than usual PA levels or overall lower PA levels compared with others were linked to more missingness in both EN and PA. Since PA was passively recorded via smartwatch, the observed negative association between same day PA and missingness likely reflects that more active days coincide with consistent device wear.

Unlike PA, EMA self-reported EN levels were not associated with EN missingness at either the within- or between-person level, indicating that individuals with lower energy—whether generally or on a given day—were not more likely to miss reporting. Interestingly, at the within-person level, missingness in smartwatch-tracked PA was higher on days they reported higher than usual EN. While unexpected, this result might reflect nuanced interactions between study and other unmeasured factors that were not accounted for by the model. Follow-up sensitivity analysis (see the “Sensitivity Analysis” section) showed that this result, while unexpected, was robust to changes in variations in the missing data models.

#### Missing at Random

As expected, the amount of phone usage was negatively associated with missingness in EMA-measured EN at both the within-person and between-person levels. More frequent phone usage than usual or compared with others was associated with less missingness in EN. However, phone usage was not related to the missingness of smartwatch-tracked PA. Self-reported sleep duration was not significantly related to missingness of either PA or EN.

We investigated the relationship between demographic variables (sex, age, employment status, self-reported depression, and alcohol consumption) and missingness in the data. First, we found that none of the demographic variables were significantly associated with missingness in EMA-measured EN. Notably, self-reported depression and age did not affect missingness. This may be because the participants were a nonclinical sample and they were all from generations familiar with smart devices, which could have minimized the impact of these variables on missingness. Conversely, people who were employed for wages showed higher missingness in smartwatch-tracked PA, suggesting that occupational demands may interfere with consistent device wear. We address the importance of considering job-related factors in influencing uHealth missing data in the “Discussion” section.

#### Burst Missingness

To account for burst missingness, we included the previous time point’s missing indicators as predictors. A positive AR coefficient indicates temporally clustered missingness. In the EN missing data model, the AR coefficient was positive and significant (*b*=0.332; 95% CrI 0.312-0.352), suggesting that if a participant missed responding to EMA once, they were more likely to miss responding the next day as well. However, the AR coefficient in the PA missingness model was not positive (*b*=–0.011; 95% CrI –0.019 to –0.003), which will be further discussed in the “Sensitivity Analysis” section. The different AR patterns between EMA-measured EN and smartwatch-tracked PA imply that missing data patterns may differ between active self-report EMA measures and passively recorded smartwatch-tracked measures.

### Sensitivity Analysis

We compared our findings from the proposed joint model (joint model 1) with other missing data handling techniques that do not explicitly incorporate missing data models: (1) listwise deletion, (2) LOCF, (3) mean imputation, (4) Bayesian FIML, (5) SAITS base, and (6) BRITS. Furthermore, to examine the sensitivity of our conclusions to the specification of the missing data model itself, we tested an alternative joint model (7), joint model 2, that extended joint model 1 by including an additional predictor—a lagged DV—into the missingness models.

#### Substantive Model

##### AR Effect

**Figure 4:**
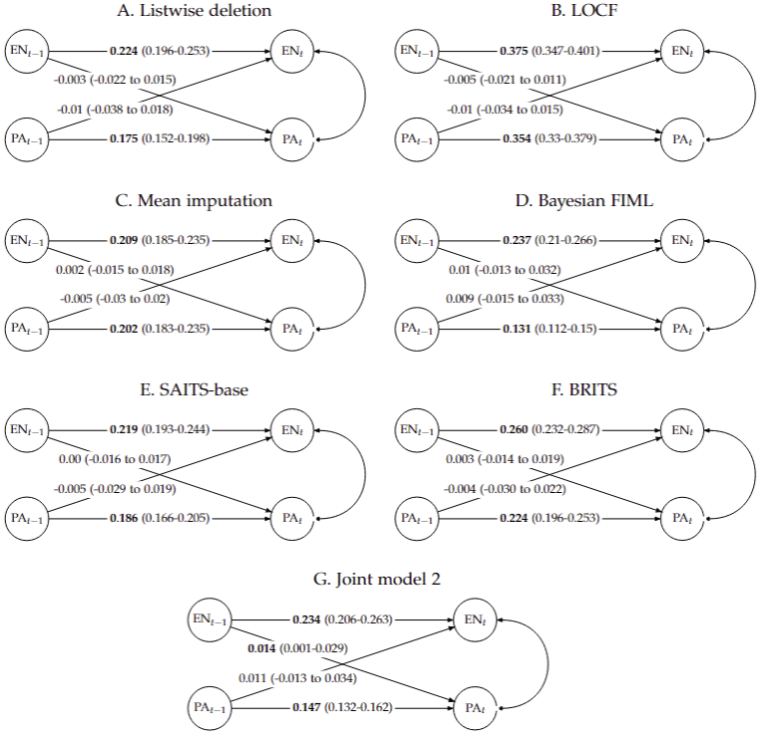
Sensitivity analysis of autoregressive (AR) and cross-regressive (CR) parameters across missing data handling methods. Each node represents EN or PA at time t or t−1. Arrows show AR and CR paths with point estimates and 95% credible intervals (within parentheses). Labels in boldface indicate significance (95% credible interval excludes 0). (A) Listwise deletion, (B) LOCF, (C) mean imputation, (D) Bayesian FIML, (E) SAITS-base, (F) BRITS, and (G) joint model 2. BRITS: Bidirectional Recurrent Imputation for Time Series; EN: energetic feeling; FIML: full information maximum likelihood; LOCF: last observation carried forward; PA: physical activity; SAITS: Self-Attention-based Imputation for Time Series.

Comparison of AR and CR effects—the key substantive model results—is summarized in Figure 4. For both PA and EN, significant positive AR coefficients were observed across all methods. The magnitudes were generally similar for EN across methods, except for LOCF, which overestimated AR parameters due to its characteristic of repeating the same value. Conversely, the AR estimates for PA were notably influenced by missing data handling methods. This appears to stem from the high incidence of MNAR missingness in PA, as discussed in the previous “Missing Data Model Result” section. As expected, AR coefficients were more similar between the Bayesian FIML and joint models than with other 2-step approaches.

##### CR Effect

In contrast to AR effects, CR coefficients—representing the dynamic interplay between energy levels and PA—were much closer to 0, thus yielding inferential conclusions that were sensitive to changes in missing data handling methods (Figure 4). Across approaches without an explicit missing data model, in models (A) to (F), CR estimates were not statistically significant. However, when joint modeling was applied (joint models 1 and 2), a significant CR effect emerged. Similar to joint model 1, joint model 2 also showed that higher energy levels on one day predicted higher PA levels on the next, demonstrating the robustness of this finding. However, the reverse CR effect (from PA to energy) was not significant in joint model 2, unlike in joint model 1.

These findings illustrate how joint modeling can uncover dynamic associations that might be obscured when missing data are not explicitly modeled. They also show how the estimation of CR effects can be influenced by the specific construction of the missing data model, highlighting the importance of sensitivity analysis across missing data model specifications.

##### Person-Level Covariate Effect

We investigated whether the baseline levels of DVs are associated with person-specific characteristics such as sex, age, employment status, depression, and alcohol consumption habit. Overall, the estimates were consistent across different methods. One exception was the effect of sex on PA baseline: although estimated sex differences from other missing data handling methods were in the same direction (ie, positive), it was significant only in 2 joint models and SAITS, where females exhibited a higher baseline PA (joint model 1: *b*=0.219, 95% CrI 0.017-0.42; joint model 2: *b*=0.226, 95% CrI 0.025-0.43; and SAITS: *b*=0.0.190, 95% CrI 0.017-0.353). This aligns with our simulation results, which are elaborated later, and indicates that while the estimates of person-level covariate effects on baseline levels are generally less sensitive than CR effects, some parameters can still be influenced by missing data handling methods.

#### Missing Data Model

The probit model results for joint model 2 are shown in Table S2 in Multimedia Appendix 2. Compared with joint model 1, here we address some robust findings and note key differences from joint model 2. Under joint model 2, the previous day’s EMA-measured EN level was not significantly associated with EN missingness, and the overall estimation on EN missingness mechanisms remained unchanged. In contrast, the previous day’s smartwatch-tracked PA level was positively associated with PA missingness (95% CrI 0.214-0.249), resulting in some shifts in parameter estimates— already noted in the “Substantive Model” subsection of “Sensitivity Analysis.”

##### Missing Not at Random

Consistent with joint model 1, joint model 2 showed that lower smartwatch-tracked PA levels were associated with higher missingness in both EMA self-reported EN and device-tracked PA measures at the within- and between-person levels, supporting the robustness of this pattern. Additionally, both models revealed an unexpected within-person effect: on days when individuals reported higher than usual EN, there was more missingness in PA measures. While unexpected, this result remained robust across different model specifications. To investigate further, we fitted another joint model with a weekend indicator to predict outcome missingness. This revealed higher PA missingness on weekends (*b*=0.093, 95% CrI 0.068-0.119), but the other main results remained unchanged.

##### Missing at Random

A higher frequency of alcohol consumption was associated with more missingness in smartwatch-tracked PA in joint model 1, but not in joint model 2. While individuals who consume alcohol more frequently may experience forgetfulness or have lifestyle choices prioritizing social activities over research participation [45], this association appeared sensitive to the missing data model specification. Another less robust finding was a sex difference in smartwatch-tracked PA missingness: females showed higher missingness in joint model 2 but not in joint model 1. This suggests that certain between-person effects—alcohol consumption and sex—may be more model-dependent and should be interpreted with extra caution.

##### Burst Missingness

In EN missingness model, similar positive AR coefficients (0.332 in joint model 1 and 0.331 in joint model 2) suggest a robust pattern of burst EN missingness: participants who missed an EMA-measured EN response were more likely to miss the following day as well. However, the AR coefficient in the PA missingness in joint model 2 (*b*=0.031, 95% CrI 0.023-0.039) differed from joint model 1. This difference suggests that burst missingness in PA is partially driven by the PA levels themselves (ie, MNAR), as well as by other unexplained autocorrelated processes. In joint model 2, we included lagged PA as a predictor, allowing the model to attribute part of the burst missingness to the autocorrelated nature of PA. As a result, the AR term—which previously captured both structured (ie, MNAR-related) and unstructured burst missingness—changed, now reflecting primarily the unstructured component of burst missingness that is unrelated to PA levels.

### Simulation Study Results

#### Simulated Substantive Model Results

To evaluate the consequences of omitting components or the entire missing data model, we conducted a simulation study designed to closely mirror the structure and characteristics of our empirical data (see Multimedia Appendix 3 for full simulation design). By anchoring the data generation on true parameter values, simulation lets us quantify bias when omitting specific components of the missing data model or entire missing data models.

Briefly, we generated complete longitudinal datasets for 200 participants over 300 days using a 2-outcome multilevel VAR (1) model, with known AR, CR, and covariate effects. Parameter values were set to approximate those estimated in the empirical analysis. We then introduced 30% missingness on both outcomes and a time-varying covariate under missing completely at random (MCAR), MAR, and MNAR mechanisms—incorporating within- and between-person predictors, individually different overall compliance rates, and burst missingness. Finally, we compared 5 analysis strategies (complete data, Bayes-FIML, and 3 joint model variants) across 100 replications. Results are summarized graphically in Figure S1 in Multimedia Appendix 3.

##### When There Were No Missing Data

Fitting the substantive model to fully observed data (“full data” condition in Figure S1 in Multimedia Appendix 3) provides a benchmark for other models. Under this ideal condition, estimations were unbiased: all closely approximate the true values. Only the covariate effects on AR were slightly underestimated, within an acceptable range.

##### Complete Omission of Missing Data Model

What happens if missing data models are not included at all? When missing data were handled solely through Bayesian FIML, estimates were severely biased across all parameters compared with models with explicit missing data models (see Bayes FIML condition in Figure S1 in Multimedia Appendix 3). These findings underscore the importance of incorporating dedicated missing data models, especially with MNAR.

##### Joint Modeling: When the Missing Data Model Was Included

When results were obtained using our proposed joint model (joint model 1; Joint DV condition in Figure S1 in Multimedia Appendix 3), which included missingness models for 2 DVs, estimation results from the joint model were almost comparable to fitting models to fully observed data despite the use of MNAR data. Notably, despite being small (0.05), the CR coefficients were accurately estimated.

##### Adding a Missing Data Model for Time-Varying Covariates

In many cases, time-varying covariates also have missingness. Should we incorporate missingness models for them, too? The short answer is: when time-varying covariates have MNAR missingness, proper missing data models can improve estimation. However, compared with joint models that accounted for missingness only in DVs, adding missing data models for covariates did not always lead to substantial changes in statistical inference. Therefore, a pragmatic approach may be to include missing data models for the DVs under thorough explorations and as guided by theories but incorporate simpler missing data handling methods on the time-varying covariates (eg, partial FIML proposed by Ji et al [26]).

##### When Clustered Missingness Was Not Accounted for

What happens when missingness is temporally clustered but AR terms to capture the burst missingness are not included in the missing data model? Our simulation shows that while omitting AR terms is not as detrimental as excluding the missing data model entirely, it can still lead to subtle but meaningful bias in certain parts of the substantive model. For detailed results and more discussion, see Multimedia Appendix 3.

#### Simulated Missing Data Model Results

The missing data model estimates under the “Joint DV” condition are shown in Table S1 in Multimedia Appendix 3. Overall, the results suggested that all parameters from the multilevel probit model were well recovered under the joint modeling framework. At the within-person level, all coefficients were estimated accurately, regardless of whether the missing mechanism was MAR or MNAR, with the absolute relative bias ranging from 0.0 to 0.08—well below the commonly accepted threshold of 0.1. This demonstrated the probit model’s adeptness in identifying both MAR and MNAR. Also, the AR coefficients were accurately estimated, indicating that the model can capture clustered missing patterns if they existed. At the between-person level, however, the coefficients were generally overestimated, with relative biases falling within a range typically considered unacceptable (0.1). Despite these biases, the coefficients still correctly captured the directions and approximate magnitudes of the true values. Thus, while person-level coefficients may lack some accuracy, they remain practically meaningful in terms of direction and magnitude.

Therefore, when used in conjunction with an appropriate substantive model, the proposed missing data model can offer useful insights into missing data mechanisms, even for MNAR scenarios. While testing for MNAR is widely recognized as challenging, using joint models may help in gaining a better understanding of these mechanisms. However, when applied to empirical data, caution is always necessary, as the true model for both the substantive and missing processes is unknown.

## Discussion

### Principal Results

We illustrated the process of fitting a multilevel VAR model to ILD, addressing potential MNAR missingness using a multilevel joint modeling framework. Our approach extends prior selection models to specifically handle intermittent missingness in ILD by incorporating within- and between-person decomposition and by capturing the temporal dependencies in the missing data model. We further conducted a sensitivity analysis comparing this approach with commonly used missing data methods, a deep leaning–based imputation technique, and alternative specifications of the missing data model—thus evaluating the robustness of findings on both substantive behavioral processes and underlying missing mechanisms.

We proposed examining the plausible missingness mechanisms through the missing data model. These insights are valuable for future mHealth and uHealth designs, as identifying patterns in missingness under certain conditions can help reduce such occurrences or address technological challenges that may disproportionately affect specific subgroups. Specifically, our analysis consistently revealed, across different missing data model specifications, that missingness in smartwatch-tracked PA and EMA-measured EN is not random but rather MNAR. That is, lower than usual PA levels, or overall lower PA levels, were associated with increased missingness in both PA and EN. This suggests that people are more likely to have missing data when they are less active, possibly because they are less mindful of wearing tracking devices during low activity. This also aligns with prior discussions in the study by Nahum-Shani et al [7], which suggest that insufficient physical energy levels may contribute to higher noncompliance rates in responding to EMA prompts or wearing wearable devices. Interestingly, yesterday’s high PA was linked to more missingness in PA today. This could be due to factors such as smartwatch battery issues—where a day of high PA leads to excessive battery consumption, leaving the smartwatch uncharged the next day and resulting in missing data. Alternatively, it could be a pattern where 1 day of high PA leads to a PA drop the following day, which in turn increases the likelihood of not wearing the smartwatch. While the PA process exhibited an AR pattern in the overall sample, excessive PA on one day can reduce the next day’s PA [46]. Confirming these potential reasons would require follow-up interviews or further investigation.

Additionally, employed individuals showed higher PA missing rates, suggesting that employment status may influence mHealth and uHealth data compliance. Job-related factors, such as demanding schedules, limited time to resolve technical issues, and stress, could affect missingness. For example, based on our research note, issues such as device malfunctions or charger problems often require contact with research staff for resolution. However, individuals with regular jobs may have fewer flexible schedules, making it difficult to address these issues. Additionally, Martinez et al [12] found that individuals in supervisory roles, who experience higher busyness, exhibited lower compliance. Researchers may need to consider these factors when designing uHealth studies.

Beyond identifying missingness mechanisms, we also examined how accounting for these mechanisms through joint modeling influenced the estimation of substantive behavioral inferences. Our substantive model included 2 key components: AR effects, capturing temporal stability, and CR effects, capturing the reciprocal influences between PA and EN. While AR effects remained consistent across different missing data handling methods, CR effects were significant only when using the joint modeling approach. The CR effects, although relatively small, reflected the reciprocal dynamics between variables and were of central interest in studies of health behaviors. Thus, this finding highlights the value of joint modeling and the importance of sensitivity analysis in assessing the robustness of parameter estimates in the presence of MNAR. Importantly, follow-up sensitivity analysis using alternative missing data model specification suggested that only the CR from EN to PA was robust to variations in the missing data model. The opposite CR effect (from PA to EN) was not. This underscored the need for sensitivity analyses with alternative missing data model specifications, even when using advanced missing data techniques such as joint modeling.

Nonetheless, our simulation study suggested that the joint modeling approach provided more reliable estimates than other methods under MNAR conditions. The proposed joint model yielded results comparable with those from fully observed data despite MNAR data. In contrast, simply fitting the substantive model alone, without explicitly modeling missingness, led to overall biased estimates. CR effects were particularly vulnerable due to their typically smaller magnitude than AR effects, resulting in low power (around 30%) when MNAR was not dealt with in the model. This aligned with our empirical results, where the CR effects were significant only in the joint models.

While joint modeling offers a principled approach grounded in theory, there has been a growing interest in mHealth and uHealth studies to use machine learning (eg, deep learning–based time series imputations) to handle missing data. Most of these approaches, including the approaches considered in our sensitivity analysis, typically handle the missingness and substantive model fitting as a 2-step process (imputation followed by model fitting), thus missing the opportunity to leverage the interdependence between the substantive and missing data models. Also, deep learning models often lack interpretability of conventional statistical models, making it difficult to explore missing mechanisms. They also require large sample sizes and extensive hyperparameter tuning, which can be a barrier in some studies. This may explain why SAITS and BRITS, in our sensitivity analysis, performed similarly to methods without explicit missing data models. While these models hold potential, their current performance may be limited by their assumptions about MCAR [42], the need for further hyperparameter tuning and larger samples, which may require additional refitment for behavioral data contexts. Nevertheless, the proposed joint framework can, in principle, be extended to deep learning architectures—allowing for simultaneous modeling for the substantive and missingness processes and thus “sharing” of information from these different data streams to strengthen the overall prediction as well as classification results.

### Limitations

In this study, we leveraged model-implied values for the substantive processes afforded by the multilevel VAR model to shed light on the missing data processes. In practice, there is no guarantee that the multilevel VAR model reflects the true change mechanisms of the substantive processes. Thus, results from modeling, including those from the missing data model, should always be interpreted with some caution. Nevertheless, our view is that the multilevel VAR model, as one of the most commonly adopted models for describing ILD processes [47], serves as a useful proxy for describing the unfolding of PA and affective processes in the study participants.

Our proposed missing data model was also limited in that we did not consider participant attrition. The missing data model used in our example mainly addressed intermittent missingness. To address dropout as well, a multinomial logit model [11] may be used as an extension to our proposed model to categorize participants’ missingness tendency into 3 nominal categories: observed, intermittent missing, and dropout, thereby providing a more comprehensive approach to handling missing data in longitudinal studies.

Additionally, our analysis was based on daily aggregated data and did not delve into specific reasons behind the missing accelerometer data, such as data transfer issues, battery drain, or the watch being powered off. Instead, we focused on the broader issue of missingness, emphasizing psychological, physical, and individual factors. Exploring reasons related to technical issues in depth would require analysis at a finer granularity, such as minute-by-minute or second-by-second data. However, this level of detail was beyond the scope of our study.

As a future direction aligned with a finer timescale analysis, it would also be beneficial to differentiate between partial missing data (eg, 2-3 hours of device wear time less than 10 hours) and complete missing data (0 hours of wear time) for PA, as there may be qualitative differences between these scenarios. Similarly, while this paper focuses on day level, a multilevel joint modeling approach could be more advantageous in studies where EMA items are measured multiple times a day. For example, the relationship between PA and EMA missingness might appear weak or inconsistent at the day level, but at a finer scale, individuals who engage in high levels of PA during EMA prompts may be less likely to respond to those prompts [9]. Finally, we note that the results of the missing data model may depend on the choice of PA measure. Notably, there is no universally accepted convention for measuring PA [48]. Researchers who measured PA using accelerometers have constructed PA metrics in several ways, including (1) the total volume of activity counts or acceleration signals accumulated throughout a specified time interval (eg, day) [49]—similar to total daily step count; (2) the mean activity counts or signals per hour or minute, which offer an average measure of activity intensity [50]; and (3) the duration of time spent in PA categorized by intensity thresholds, such as moderate to vigorous PA [51]. Depending on the PA measure used, the definition of missing or valid data can vary, leading to potentially divergent model estimation results [52].

Additionally, PA data are often positively skewed, with a concentration of data at the lower end of the scale [53]. This deviation from normality may pose a potential limitation, as joint modeling approaches typically rely on the normality assumption [15]. While addressing this limitation is beyond the scope of this study, future research should consider modeling strategies that accommodate nonnormal data distribution, such as transformation or using a gamma link function [53].

### Conclusions

This study enriches our understanding of missing data mechanisms and strategies for managing them in ILD. Key contributions include the following: First, incorporating missing data mechanisms into the model not only improves the properties of the estimation results but also provides a nuanced understanding of missing data mechanisms. Understanding the reasons for missing data holds significant value for several reasons. If the reasons are within the researcher’s control, it can provide insights into how to collect data in future studies. Detecting contextual factors related to missingness can be used to pinpoint ways to improve participants’ engagement. Additionally, understanding the mechanisms behind missing data can sometimes provide deeper insights into the variables themselves, thus hastening researchers’ and practitioners’ understanding of the phenomenon under study.

Second, we highlight the importance of sensitivity analysis. Evaluating the sensitivity and robustness of specific model aspects in relation to missing data provides crucial insights for informed medical decisions based on model outcomes. If the results exhibit sensitivity to methodological changes, it necessitates a thoughtful interpretation of the conclusions regarding their effects and suggests a potential need for further research.

Third, we demonstrated the effectiveness of our approach using simulated data that closely resemble actual datasets. We showed that our multilevel joint modeling is effective even with ordinal variables, such as Likert scale variables, and could reveal even strong MNAR mechanisms, as evidenced by the large coefficients observed in the empirical results. Furthermore, AR terms in the missing data model were able to capture frequently observed temporal dependency in missingness in ILD. However, it is important to note that the outcome of joint modeling may differ based on the chosen missing data model specification and still can give biased results if the specified model is not correct. Therefore, we strongly recommend performing sensitivity analysis with multiple missing data models to ensure the robustness of findings and avoid overreliance on a single model.

In summary, our findings highlight the importance of appropriately handling nonignorable missing data, particularly through multilevel joint modeling, followed by sensitivity analysis. This workflow will improve the validity of substantive conclusions and enhance our understanding of the underlying missing data mechanisms, which can inform better study designs and intervention strategies. While our analysis focused on a limited set of plausible variables, researchers would benefit from exploring a broader array of the psychological and behavioral factors that may influence compliance in mHealth studies. We hope this study serves as a framework for future investigations.

## Appendix

Multimedia Appendix 1 Code for empirical analysis.

Multimedia Appendix 2 Missing data model results.

Multimedia Appendix 3 Simulation study materials.

## Abbreviations

AR: autoregressive
BRITS: Bidirectional Recurrent Imputation for Time Series
CR: cross-regressive
CrI: credible interval
DV: dependent variable
EMA: ecological momentary assessment
EN: energetic feeling
FIML: full information maximum likelihood
ILD: intensive longitudinal data
LOCF: last observation carried forward
MAR: missing at random
MCAR: missing completely at random
mHealth: mobile health
MNAR: missing not at random
PA: physical activity
PMM: pattern mixture model
SAITS: Self-Attention-based Imputation for Time Series
SPM: shared parameter model
SWaN: Sleep Wake and Nonwear algorithm
uHealth: ubiquitous health
VAR: vector autoregressive

## References

[1] Fuller D, Colwell E, Low J, Orychock K, Tobin MA, Simango B, et al (2020). Reliability and validity of commercially available wearable devices for measuring steps, energy expenditure, and heart rate: systematic review. JMIR Mhealth Uhealth.

[2] Dunton GF, Dzubur E, Intille S (2016). performance test of a real-time sensor-informed context-sensitive ecological momentary assessment to capture physical activity. J Med Internet Res.

[3] Dao KP, De Cocker K, Tong HL, Kocaballi AB, Chow C, Laranjo L (2021). delivered ecological momentary interventions based on ecological momentary assessments to promote health behaviors: systematic review and adapted checklist for reporting ecological momentary assessment and intervention studies. JMIR Mhealth Uhealth.

[4] Dunton GF, Rothman AJ, Leventhal AM, Intille SS (2021). How intensive longitudinal data can stimulate advances in health behavior maintenance theories and interventions. Transl Behav Med.

[5] Gates K, Chow S, Molenaar P (2023). Intensive Longitudinal Analysis of Human Processes. First edition.

[6] Wang S, Yang C, Brown D, Cheng A, Kwan MYW (2025). compliance with ecological momentary assessment in movement behavior research among adolescents and emerging adults: systematic review. JMIR Mhealth Uhealth.

[7] Nahum-Shani I, Shaw SD, Carpenter SM, Murphy SA, Yoon C (2022). Engagement in digital interventions. Am Psychol.

[8] van Berkel N, Goncalves J, Hosio S, Sarsenbayeva Z, Velloso E, Kostakos V (2020). Overcoming compliance bias in self-report studies: a cross-study analysis. Int J Hum-Comput Stud.

[9] Ponnada A, Li J, Wang SD, Wang W, Dunton GF, Intille SS (2022). biases in microinteraction ecological momentary assessment (μEMA) non-response. Proc ACM Interact Mob Wearable Ubiquitous Technol.

[10] Stone AA, Schneider S, Smyth JM (2023). Evaluation of pressing issues in ecological momentary assessment. Annu Rev Clin Psychol.

[11] Yuan C, Hedeker D, Mermelstein R, Xie H (2020). A tractable method to account for high-dimensional nonignorable missing data in intensive longitudinal data. Stat Med.

[12] Martinez GJ, Mattingly SM, Robles-Granda P, Saha K, Sirigiri A, Young J, et al (2021). Predicting participant compliance with fitness tracker wearing and ecological momentary assessment protocols in information workers: observational study. JMIR Mhealth Uhealth.

[13] Li Y, Oravecz Z, Ji L, Chow S (2024). multiple imputation with factor scores: a practical approach for handling simultaneous missingness across items in longitudinal designs. Multivariate Behav Res.

[14] Tang N, Chow S, Ibrahim JG, Zhu H (2017). Bayesian sensitivity analysis of a nonlinear dynamic factor analysis model with nonparametric prior and possible nonignorable missingness. Psychometrika.

[15] Little R, Rubin D (2002). Statistical Analysis With Missing Data. 2nd Edition.

[16] Little RJA (1993). Pattern-mixture models for multivariate incomplete data with covariates. J Am Stat Assoc.

[17] Enders C (2022). Applied Missing Data Analysis. Second edition.

[18] Savalei V, Bentler PM (2009). A two-stage approach to missing data: theory and application to auxiliary variables. Struct Equation Model Multidisciplinary J.

[19] Albert PS (2019). Shared random parameter models: a legacy of the biostatistics program at the National Heart, Lung, and Blood Institute. Stat Med.

[20] Wu MC, Carroll RJ (1988). Estimation and comparison of changes in the presence of informative right censoring by modeling the censoring process. Biometrics.

[21] Enders CK (2023). Missing data: an update on the state of the art. Psychol Methods.

[22] Little RJA (1995). Modeling the drop-out mechanism in repeated-measures studies. J Am Stat Assoc.

[23] Yue Xu S, Nelson S, Kerr J, Godbole S, Patterson R, Merchant G, et al (2018). Statistical approaches to account for missing values in accelerometer data: applications to modeling physical activity. Stat Methods Med Res.

[24] Orstad SL, Gerchow L, Patel NR, Reddy M, Hernandez C, Wilson DK, et al (2021). Defining valid activity monitor data: a multimethod analysis of weight-loss intervention participants' barriers to wear and first 100 days of physical activity. Informatics (MDPI).

[25] Yucel RM (2008). Multiple imputation inference for multivariate multilevel continuous data with ignorable non-response. Phil Trans R Soc A.

[26] Ji L, Chen M, Oravecz Z, Cummings EM, Lu Z, Chow S (2020). A Bayesian vector autoregressive model with nonignorable missingness in dependent variables and covariates: development, evaluation, and application to family processes. Struct Equ Modeling.

[27] McNeish D, Somers JA, Savord A (2024). Dynamic structural equation models with binary and ordinal outcomes in Mplus. Behav Res Methods.

[28] Wang S, Intille S, Ponnada A, Do B, Rothman A, Dunton G (2022). Investigating microtemporal processes underlying health behavior adoption and maintenance: protocol for an intensive longitudinal observational study. JMIR Res Protoc.

[29] John D, Tang Q, Albinali F, Intille S (2019). An open-source monitor-independent movement summary for accelerometer data processing. J Meas Phys Behav.

[30] Gelman A (2004). Parameterization and Bayesian modeling. J Am Stat Assoc.

[31] Troiano RP, Berrigan D, Dodd KW, Mâsse LC, Tilert T, McDowell M (2008). Physical activity in the United States measured by accelerometer. Med Sci Sports Exerc.

[32] Garriga A, Sempere-Rubio N, Molina-Prados MJ, Faubel R (2021). Impact of seasonality on physical activity: a systematic review. Int J Environ Res Public Health.

[33] Thomadakis C, Meligkotsidou L, Pantazis N, Touloumi G (2020). Misspecifying the covariance structure in a linear mixed model under MAR drop‐out. Stat Med.

[34] Chen G, Tsurumi H (2011). Probit and logit model selection. Commun Stat Theory Methods.

[35] Hahn E, Soyer R (2008). Probit versus logit models: the role of the link function in the multivariate realm. Technical Report TR-2008-17. The Institute for Integrating Statistics in Decision Sciences, The George Washington University.

[36] Cho YW, Chow S-M, Marini CM, Martire LM (2024). Multilevel latent differential structural equation model with short time series and time-varying covariates: a comparison of frequentist and Bayesian estimators. Multivariate Behav Res.

[37] Oravecz Z, Tuerlinckx F, Vandekerckhove J (2011). A hierarchical latent stochastic differential equation model for affective dynamics. Psychol Methods.

[38] Chen M, Chow S, Oravecz Z, Ferrer E (2023). Fitting Bayesian stochastic differential equation models with mixed effects through a filtering approach. Multivariate Behav Res.

[39] Kruschke JK (2021). Bayesian analysis reporting guidelines. Nat Hum Behav.

[40] Hamaker EL, Asparouhov T, Brose A, Schmiedek F, Muthén B (2018). At the frontiers of modeling intensive longitudinal data: dynamic structural equation models for the affective measurements from the COGITO study. Multivariate Behav Res.

[41] Asparouhov T, Hamaker EL, Muthén B (2017). Dynamic structural equation models. Struct Equ Modeling.

[42] Du W, Côté D, Liu Y (2023). SAITS: self-attention-based imputation for time series. Expert Syst Appl.

[43] Cao W, Wang D, Li J, Zhou H, Li Y, Li L (2018). BRITS: bidirectional recurrent imputation for time series.

[44] Du W (2023). PyPOTS: A Python Toolbox for Data Mining on Partially-Observed Time Series. arXiv. Preprint posted online on May 30, 2023.

[45] Wetherill RR, Fromme K (2011). Acute alcohol effects on narrative recall and contextual memory: an examination of fragmentary blackouts. Addict Behav.

[46] Funabashi D, Dobashi S, Sameshima K, Sagayama H, Nishijima T, Matsui T (2024). Acute vigorous exercise decreases subsequent nonexercise physical activity and body temperature linked to weight gain. Med Sci Sports Exerc.

[47] Lafit G, Meers K, Ceulemans E (2022). A systematic study into the factors that affect the predictive accuracy of multilevel VAR(1) models. Psychometrika.

[48] Migueles JH, Cadenas-Sanchez C, Ekelund U, Delisle NC, Mora-Gonzalez J, Löf M, Labayen I, et al (2017). Data collection and processing criteria to assess physical activity and other outcomes: a systematic review and practical considerations. Sports Med.

[49] Choudhury R, Park J, Banarjee C, Coca MG, Fukuda DH, Xie R, et al (2024). Associations between monitor-independent movement summary (MIMS) and fall risk appraisal combining fear of falling and physiological fall risk in community-dwelling older adults. Front Aging.

[50] Do B, Hedeker D, Wang W, Mason TB, Belcher BR, Miller KA, et al (2024). Investigating the day-level associations between affective variability and physical activity using ecological momentary assessment. Psychol Sport Exerc.

[51] Colley RC, Tremblay MS (2011). Moderate and vigorous physical activity intensity cut-points for the Actical accelerometer. J Sports Sci.

[52] Baroudi L, Zernicke RF, Tewari M, Carlozzi NE, Choi SW, Cain SM (2025). Using wear time for the analysis of consumer-grade wearables’ data: case study using Fitbit data. JMIR Mhealth Uhealth.

[53] Baldwin SA, Fellingham GW, Baldwin AS (2016). Statistical models for multilevel skewed physical activity data in health research and behavioral medicine. Health Psychol.

