# Supplementary material for: Within- and Between-Individual Compliance in Mobile Health: Joint Modeling Approach to Nonrandom Missingness in an Intensive Longitudinal Observational Study
Source: JMIR Mhealth Uhealth. 2025 Oct 30;13:e65350. doi: 10.2196/65350 (PMC12616189; doi:10.2196/65350)
Supplement: Multimedia Appendix 1 [file mhealth_v13i1e65350_app1.docx]

**Multimedia Appendix 1**

DATA: FILE = empiricaldata.csv;

VARIABLE:

Names = ID time season EN sleep phone PA weartime e_idx p_idx

sex age employ depression alcohol; ! List of all variables in the dataset

USEVAR = ID time season EN sleep phone PA weartime e_idx p_idx

sex age employ depression alcohol; ! Variables used in the model

! Variable Descriptions:

! time: study days

! season: season indicator

! EN: daily self-reported energetic feeling

! sleep: daily sleep duration

! phone: daily count of phone unlock

! PA: daily smartwatch-tracked physical activity

! weartime: daily device wear time

! e_idx: a binary indicator of missingness of daily energetic feeling (1: missing, 0: observed)

! p_idx: a binary indicator of missingness of daily PA (1: missing, 0: observed)

! sex, age, employ, depression, alcohol: person-level covariates

LAGGED = EN(1) PA(1) e_idx(1) p_idx(1); ! 1st-order lag for DVs and missingness indicators

CLUSTER = ID; ! Multilevel clustering by participant

WITHIN = season; ! within-person covariates not used in the between-person level

BETWEEN = sex age employ depression alcohol; ! between-person covariates

MISSING = ALL(-999); ! Missing value code in empiricaldata.csv

TINTERVAL = time(1); ! Specifies that time is structured at daily intervals

CATEGORICAL = EN e_idx p_idx; ! Specifies binary/categorical variables for probit link

DEFINE:

CENTER sex age employ depression alcohol sleep phone weartime (grandmean);

! Center continuous covariates at grand mean to aid interpretability

ANALYSIS:

TYPE = TWOLEVEL RANDOM; ! Two-level modeling with random slopes

ESTIMATOR = BAYES; ! Bayesian estimation

PROC = 2; ! Number of MCMC chains

BITER = (3000); ! Number of MCMC iterations per chain (min 3000)

MODEL:

%WITHIN% ! within-person level model starts

! Covariates:

sleep weartime season; ! Specifying distributional assumptions for dealing with missing data in exogeneous variables

EN ON sleep; ! Predict daily energetic feeling from sleep

PA ON weartime season; ! Predict PA from wear time and seasonal variation

! Substantive Model (VAR):

ar_EN | EN ON EN&1; ! random AR for energetic feeling

ar_PA | PA ON PA&1; ! random AR for physical activity

EN_on_PA1 | EN on PA&1; ! random CR: yesterday’s PA predicting today’s EN

PA_on_EN1 | PA on EN&1; ! random CR: yesterday’s EN predicting today’s PA

! Missing Data Model (Probit):

e_idx ON e_idx&1 EN PA sleep phone; ! Missingness in EN predicted by its past missing indicator, DVs, and covariates.

p_idx ON p_idx&1 EN PA sleep phone; ! Missingness in PA predicted by its past missing indicator, DVs, and covariates.

! Allowing residual correlations among outcomes and missingness indicators

EN PA e_idx p_idx WITH PA e_idx p_idx;

%BETWEEN% ! Between-person level model starts

! Covariates:

sleep phone weartime sex age employ depression alcohol; ! Specifying distributional assumptions for dealing with missing data in exogeneous variables

EN ON sleep sex age employ depression alcohol; ! Between-person predictors of average EN

PA ON weartime sex age employ depression alcohol; ! Between-person predictors of average PA

! Substantive Model’s Random Slopes Specified in %WITHIN% part (automatically estimated)

ar_EN;

ar_PA;

EN_on_PA1;

PA_on_EN1;

! Missing Data Model (Probit) at between-person level

e_idx ON EN PA sleep phone sex age employ depression alcohol;

p_idx ON EN PA sleep phone sex age employ depression alcohol;

! Correlated random effects among outcomes and missingness

EN PA ar_EN-PA_on_EN1 e_idx p_idx WITH

PA ar_EN-PA_on_EN1 e_idx p_idx ;

OUTPUT: TECH1 TECH8; ! Request tech output for model specification and convergence diagnostics

PLOT: TYPE = PLOT3; ! Plotting for Bayesian posterior distributions and diagnostics

NOTE: 'season' is a time-varying covariate that varies only within-person (i.e., no between-person variance), and is therefore explicitly specified in the WITHIN list. In contrast, variables like 'sleep' and 'phone' exhibit both within- and between-person variability. To allow Mplus to apply “latent group-mean centering” to these predictors, they are not specified under either WITHIN or BETWEEN. This lets Mplus estimate their within-person effects at Level 1 while modeling between-person variability at Level 2.

In our study, Bayesian estimation was used for all models, employing the default settings of Mplus. Specifically, the Bayesian estimator in Mplus utilizes a Markov Chain Monte Carlo (MCMC) algorithm based on the Gibbs sampler. By default, Mplus runs two parallel chains, each initialized with different starting values to aid in convergence diagnostics. The default number of iterations is 50,000 per chain, with the first 25,000 iterations used as burn-in and discarded. Conjugate priors are assigned automatically based on model structure and parameter type. Convergence is assessed using the Potential Scale Reduction (PSR) factor, with values below 1.1 indicating satisfactory convergence. Unless otherwise specified, point estimates are obtained from the posterior median, and 95% credible intervals are derived from the 2.5th and 97.5th percentiles of the posterior distribution. For more details about the priors, see [1].

1. Asparouhov T, Muthén B. Bayesian analysis using Mplus: Technical implementation. 2010. Available from: https://www.statmodel.com/download/Bayes2.pdf
