# Supplementary material for: Within- and Between-Individual Compliance in Mobile Health: Joint Modeling Approach to Nonrandom Missingness in an Intensive Longitudinal Observational Study
Source: JMIR Mhealth Uhealth. 2025 Oct 30;13:e65350. doi: 10.2196/65350 (PMC12616189; doi:10.2196/65350)
Supplement: Multimedia Appendix 2 [file mhealth_v13i1e65350_app2.docx]

Multimedia Appendix 2

Table S1. Empirical missing data model result from joint model 1.

| Level | Outcome | Predictor | Est. | 95% Credible Interval (CI) | | |
| --- | --- | --- | --- | --- | --- | --- |
|  |  |  |  | 2.5% | 97.5% | Zero Not Included |
| **Within-person** |  |  |  |  |  |  |
|  | **EN Missing_t_** |  |  |  |  |  |
|  |  | EN Missing_t-1_ | 0.332 | 0.312 | 0.352 | * |
|  |  | EN | -0.052 | -0.115 | 0.004 |  |
|  |  | PA | -0.128 | -0.147 | -0.108 | * |
|  |  | Sleep | 0.007 | -0.001 | 0.016 |  |
|  |  | Phone | -0.138 | -0.158 | -0.117 | * |
|  | **PA Missing_t_** |  |  |  |  |  |
|  |  | PA Missing_t-1_ | -0.027 | -0.037 | -0.018 | * |
|  |  | EN | 0.359 | 0.213 | 0.543 | * |
|  |  | PA | -1.523 | -1.558 | -1.441 | * |
|  |  | Sleep | 0 | -0.005 | 0.006 |  |
|  |  | Phone Usage | -0.003 | -0.014 | 0.009 |  |
| **Between-person** |  |  |  |  |  |  |
|  | **EN Missing** |  |  |  |  |  |
|  |  | EN | 0.031 | -0.929 | 0.971 |  |
|  |  | PA | -0.379 | -0.564 | -0.205 | * |
|  |  | Sleep | -0.084 | -0.181 | 0.011 |  |
|  |  | Phone Usage | -0.19 | -0.297 | -0.083 | * |
|  |  | Sex | -0.041 | -0.282 | 0.197 |  |
|  |  | Age | 0.009 | -0.037 | 0.052 |  |
|  |  | Employed | 0.139 | -0.176 | 0.446 |  |
|  |  | Depression | 0.055 | -0.44 | 0.527 |  |
|  |  | Alcohol | 0.053 | -0.032 | 0.14 |  |
|  | **PA Missing** |  |  |  |  |  |
|  |  | EN | -0.022 | -0.248 | 0.214 |  |
|  |  | PA | -0.959 | -1.094 | -0.839 | * |
|  |  | Sleep | -0.015 | -0.043 | 0.008 |  |
|  |  | Phone Usage | 0.002 | -0.026 | 0.03 |  |
|  |  | Sex | 0.199 | -0.002 | 0.4 |  |
|  |  | Age | 0.001 | -0.031 | 0.033 |  |
|  |  | Employed | 0.36 | 0.148 | 0.574 | * |
|  |  | Depression | -0.054 | -0.301 | 0.184 |  |
|  |  | Alcohol | 0.06 | 0.001 | 0.122 | * |

*Note*. EN: energetic feelings; PA: physical activity; $\text{EN Missing}_{t}$ and $\text{PA Missing}_{t}$: missing data indicator at time $t$, with 1: unobserved (missing), 0: observed; Sleep: self-reported sleep duration; Phone Usage: the number of times the phone screen was unlocked; Sex: 1: female, 0: male; Employed: 1: Employed for wages, 0: no; Depression: 1: yes, 0: no; Alcohol: the number of days per week on which a drink is consumed; The 95% CI’s 2.5% and 97.5% denote the 2.5th and 97.5th percentiles of the parameter’s posterior distribution. An asterisk (*) was marked when the 95% CI did not include zero.

Table S2. Empirical missing data model result from joint model 2.

| Level | Outcome | Predictor | Est. | 95% Credible Interval (CI) | | |
| --- | --- | --- | --- | --- | --- | --- |
|  |  |  |  | 2.5% | 97.5% | Zero Not Included |
| **Within-person** |  |  |  |  |  |  |
|  | **EN Missing_t_** |  |  |  |  |  |
|  |  | EN Missing_t-1_ | 0.331 | 0.311 | 0.350 | * |
|  |  | EN | -0.028 | -0.108 | 0.031 |  |
|  |  | PA | -0.131 | -0.150 | -0.111 | * |
|  |  | EN_t-1_ | -0.001 | -0.030 | 0.031 |  |
|  |  | Sleep | 0.007 | -0.001 | 0.016 |  |
|  |  | Phone | -0.138 | -0.159 | -0.117 | * |
|  | **PA Missing_t_** |  |  |  |  |  |
|  |  | PA Missing_t-1_ | 0.031 | 0.023 | 0.039 | * |
|  |  | EN | 0.126 | 0.075 | 0.213 | * |
|  |  | PA | -1.558 | -1.557 | -1.540 | * |
|  |  | PA_t-1_ | 0.232 | 0.214 | 0.249 | * |
|  |  | Sleep | 0.001 | -0.004 | 0.005 |  |
|  |  | Phone Usage | -0.005 | -0.015 | 0.006 |  |
| **Between-person** |  |  |  |  |  |  |
|  | **EN Missing** |  |  |  |  |  |
|  |  | EN | -0.028 | -0.984 | 1.071 |  |
|  |  | PA | -0.379 | -0.585 | -0.222 | * |
|  |  | Sleep | -0.088 | -0.179 | 0.001 |  |
|  |  | Phone Usage | -0.189 | -0.296 | -0.078 | * |
|  |  | Sex | -0.038 | -0.290 | 0.216 |  |
|  |  | Age | 0.008 | -0.038 | 0.053 |  |
|  |  | Employed | 0.151 | -0.172 | 0.436 |  |
|  |  | Depression | 0.034 | -0.515 | 0.580 |  |
|  |  | Alcohol | 0.054 | -0.034 | 0.147 |  |
|  | **PA Missing** |  |  |  |  |  |
|  |  | EN | 0.116 | -0.249 | 0.195 |  |
|  |  | PA | -0.907 | -1.038 | -0.787 | * |
|  |  | Sleep | -0.013 | -0.036 | 0.007 |  |
|  |  | Phone Usage | 0.002 | -0.024 | 0.028 |  |
|  |  | Sex | 0.197 | 0.005 | 0.388 | * |
|  |  | Age | 0.003 | -0.027 | 0.033 |  |
|  |  | Employed | 0.345 | 0.150 | 0.546 | * |
|  |  | Depression | -0.054 | -0.297 | 0.175 |  |
|  |  | Alcohol | 0.056 | -0.003 | 0.115 |  |

*Note*. EN: energetic feelings; PA: physical activity; $\text{EN Missing}_{t}$ and $\text{PA Missing}_{t}$: missing data indicator at time $t$, with 1: unobserved (missing), 0: observed; Sleep: self-reported sleep duration; Phone Usage: the number of times the phone screen was unlocked; Sex: 1: female, 0: male; Employed: 1: Employed for wages, 0: no; Depression: 1: yes, 0: no; Alcohol: the number of days per week on which a drink is consumed; The 95% CI’s 2.5% and 97.5% denote the 2.5th and 97.5th percentiles of the parameter’s posterior distribution. An asterisk (*) was marked when the 95% CI did not include zero.
