# Supplementary material for: Within- and Between-Individual Compliance in Mobile Health: Joint Modeling Approach to Nonrandom Missingness in an Intensive Longitudinal Observational Study
Source: JMIR Mhealth Uhealth. 2025 Oct 30;13:e65350. doi: 10.2196/65350 (PMC12616189; doi:10.2196/65350)
Supplement: Multimedia Appendix 3 [file mhealth_v13i1e65350_app3.docx]

**Multimedia Appendix 3**

In addition to the empirical analysis, we conducted a simulation study to evaluate the effectiveness of proposed joint modeling approaches. The process unfolded in three steps:

1. **Complete Dataset**: We generated complete datasets using a multilevel VAR model. All model parameters’ true values were known (i.e., set by researchers to mirror the parameter estimates obtained with empirical data).
2. **Introducing Missingness**: Missing data were introduced to the complete dataset based on a specified missing data model.
3. **Model Fitting**: We fitted the data-generating model using five modeling methods:
   1. Full Data: Ideal case with no missing data (benchmark)
   2. Bayes FIML: No missing data model; handled missingness via Bayesian FIML
   3. Joint DV: Joint model with missing data models for two outcomes
   4. Joint TVcov: Joint DV + missing data model for a time-varying covariate
   5. Joint NoAR: Joint DV without AR terms in the missing data models

After conducting these procedures across 100 replications, we compared the estimation results across missing data handling approaches.

#### Complete Data Generation

Two fully observed DVs were generated based on a multilevel VAR(1) model, mirroring the empirical data for 200 participants with repeated measures over 300 days. Each individual has baseline levels for both DVs, predicted by their previous time point values, capturing AR and CR effects. A time-varying covariate was also included to enhance the generalizability of our simulation. Individual differences (i.e., random effect) in baselines, AR and CR parameters, and effects of the time-varying covariate were incorporated, and were predicted by a between-person covariate. Additionally, one DV was transformed into a 5-point Likert scale to reflect ordinal data commonly used in behavioral research.

The technical details are as follows: Two fully observed variables, *y*_1,_*_i_*_,_*_t_* and *y*_2,_*_i_*_,_*_t_*, were generated based on a bivariate multilevel VAR(1) model outlined below. Mirroring the empirical data, *i* ranged from 1 to 200 (representing 200 participants), and *t* ranged from 1 to 300 (representing 300 days).

*y*_1,_*_i_*_,_*_t_* = *µ*_1,_*_i_* + *a*_1,_*_i_y*_1,_*_i_*_,_*_t−_*_1_ + *b*_1,_*_i_y*_2,_*_i_*_,_*_t−_*_1_ + *c*_1,_*_i_*TVcov*_i_*_,_*_t_* + *u*_1,_*_i_*_,_*_t_* **(1)**

*y*_2,_*_i_*_,_*_t_* = *µ*_2,_*_i_* + *b*_2,_*_i_y*_1,_*_i_*_,_*_t−_*_1_ + *a*_2,_*_i_y*_2,_*_i_*_,_*_t−_*_1_ + *c*_2,_*_i_*TVcov*_i_*_,_*_t_* + *u*_2,_*_i_*_,_*_t_* **(2)**

where the individual-specific parameter vectors are modeled as:

Θ*_i_* = Θ + Γ*x*_1,_*_i_* + *ϵ_i_* **(3)**

for Θ*_i_* ∈ {*µ*_1,_*_i_*, *µ*_2,_*_i_*, *a*_1,_*_i_*, *a*_2,_*_i_*, *b*_1,_*_i_*, *b*_2,_*_i_*, *c*_1,_*_i_*, *c*_2,_*_i_*}. Here, *µ*_1,_*_i_* and *µ*_2,_*_i_* represent the baseline of *y*_1,_*_i_*_,_*_t_* and *y*_2,_*_i_*_,_*_t_* for the *i_th_* individual, respectively, reflecting the levels to which the variables return in the long run. Both DVs were predicted by their previous time point values, *y*_1,_*_i_*_,_*_t−_*_1_, *y*_2,_*_i_*_,_*_t−_*_1_, capturing AR and CR effects. Additionally, a time-varying covariate predicting DVs, TVcov*_i_*_,_*_t_*, following a standard normal distribution, was included in the model to enhance the generalizability of our simulation.

To reflect the multilevel structure, individual differences in the baselines (*µ*_1,_*_i_*, *µ*_2,_*_i_*), AR parameters (*a*_1,_*_i_*, *a*_2,_*_i_*), CR parameters (*b*_1,_*_i_*, *b*_2,_*_i_*), and effects of the time- varying covariate (*c*_1,_*_i_*, *c*_2,_*_i_*) were incorporated into the model. These individual differences in parameters were predicted by a person-specific covariate, *x*_1,_*_i_*, which follows a standard normal distribution. In Equation [3](#_bookmark8), Θ represents the population-level parameter vector, Γ represents the vector of effects of the person- specific covariate *x*_1,_*_i_*.

#### Missing Data Generation

After generating the fully observed data, we set 30% of the observations for the two DVs and a time-varying covariate as missing, reflecting common missing rates in EMA studies [27]. To implement this, we used multilevel probit models to generate binary missing indicators (0: observed, 1: missing) for three variables. Importantly, the generated missing data patterns were designed to mimic those observed in the empirical dataset. Specifically:

1. Accounting for both within- and between-person sources of missingness, missing data models were constructed at both levels.
2. Reflecting individual differences in compliance, we used correlated random intercepts.
3. For various missing scenarios, we implemented MNAR, MAR, and MCAR mechanisms.
4. To capture burst missingness, we incorporated AR terms.
5. Coefficients were selected to encompass extreme values in empirical analysis.

We implemented this by generating binary missing indicators (0: observed, 1: missing) for three variables—two DVs and a time-varying covariate—using multilevel probit models. For person *i*, the binary missing indicator of the *k*th variable at time *t* (*R_k_*_,_*_i_*_,_*_t_*) is 1 if person *i*’s latent continuous tendency to exhibit missingness for the *k*th variable at time *t* (*Z_k_*_,_*_i_*_,_*_t_*) exceeds a threshold (*τ*) for categorizing *Z_k_*_,_*_i_*_,_*_t_*; otherwise (if *Z_k_*_,_*_i_*_,_*_t_ < τ*), *R_k_*_,_*_i_*_,_*_t_* is 0. The latent missingness tendency, *Z_k_*_,_*_i_*_,_*_t_* was specified to vary within- and between-individuals following Equations [4](#_bookmark9) and [5](#_bookmark10), respectively.

Within-person Level

*Z_k,i,t_* = *ϕ_01,k,i_* + AR(*Z_k,i,t-1_ − ϕ_01,k,i_* ) + *ϕ_nmar_*_1_*y_k_*_,_*_i_*_,_*_t_* + *ϕ_mar_*_1_TVcov*_i,t_* **(4)**

Between-person Level

*ϕ_01,k,i_* = *ϕ_01,k_* + *ϕ_nmar_*_2_*yMean_k,i_* + *ϕ_mar_*_2_TVcov*_i_* + *ϕ_mar_*_3_ *x*2*_i_* + *ϵ_1,i_*  **(5)**

At the within-person level, each missingness likelihood for DVs was predicted by the previous time point’s missing likelihood (*Z_k_*_,_*_i_*_,_*_t−_*_1_; to incorporate an AR term), the level of the DV itself (*y_k_*_,_*_i_*_,_*_t_*; to implement an MNAR mechanism), and one time-varying covariate (TVcov*_i_*_,_*_t_*; to implement a MAR mechanism). Similarly, for the missing likelihood of the time-varying covariate, the previous time point’s missing likelihood was included as a predictor.

To accommodate individually varying missing or adherence rates, we allowed intercepts of three probit regressions to be individually different. Thus, at the between-person level, the intercepts (*ϕ*_01,_*_k_*_,_*_i_*) were further explained by the person means of the DV itself (*yMean_k_*_,_*_i_*) and the time-varying covariate (TVcov*_i_*), and an additional person-level covariate (*x*2*_i_*; e.g., age), which follows a standard normal distribution. Since all predictors were centered at zero, intercepts determine the overall percentage of missing across all participants, with lower values associated with lower missing rates. Intercepts were set to create an overall 30% missing. Coefficients of predictors (*ϕ_mnar_*_1_, *ϕ_mnar_*_2_, *ϕ_mar_*_1_, *ϕ_mar_*_2_, *ϕ_mar_*_3_) denote how the latent tendency of missingness was influenced by specific predictors. Finally, the probability of the missing indicator *R_k_*_,_*_i_*_,_*_t_* being 1 was modeled based on the latent continuous missing tendency *Z_k_*_,_*_i_*_,_*_t_* as follows [28]: *P*(*R_k_*_,_*_i_*_,_*_t_* = 1) = *P*(*Z_k_*_,_*_i_*_,_*_t_ > τ*) = 1 *− F*(*τ − Z_k_*_,_*_i_*_,_*_t_*), where *F*(.) denotes the cumulative probability function of a standard normal distribution.

**Figure S1.** Simulated VAR model results across missing data handling methods.


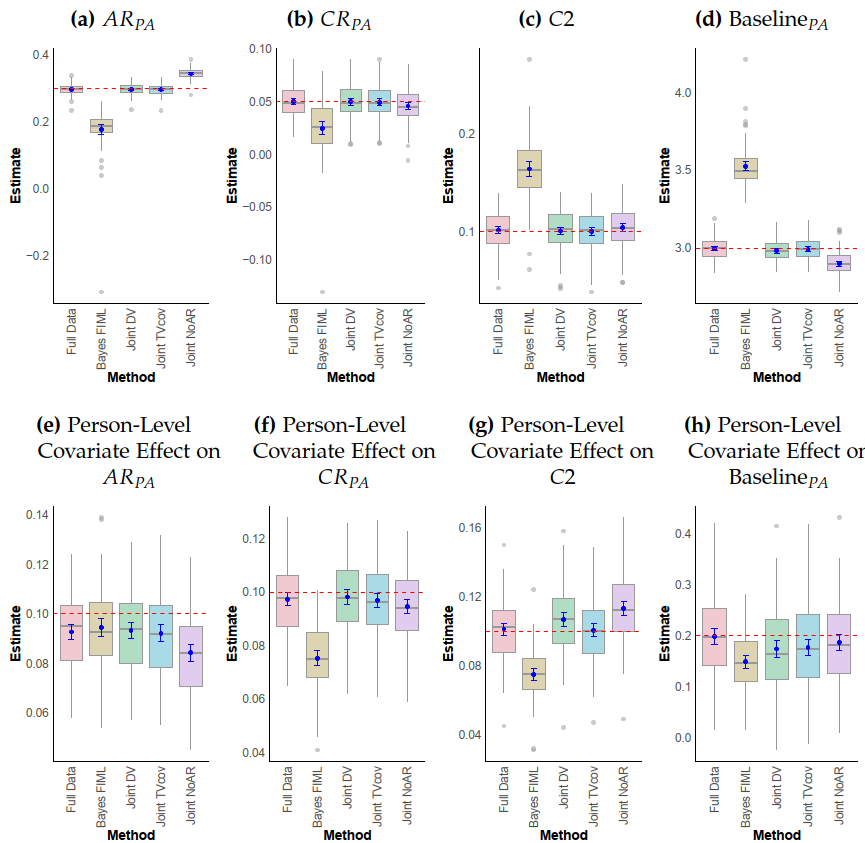


*Note*. Plots (a)-(d):performances of missing data handling methods in estimating the key parameters (AR, CR, time-varying covariate effects, and baselines). Plots (e)-(h): estimates of the effect of the person-level covariate on the key parameters. Each box plot illustrates the distribution of the estimates of each parameter (from 100 replications), including median, IQR, and outliers. The red dashed line indicates the true value. Each box plot displays mean (blue dot) and error bar (2.5th and 97.5th percentiles of the point estimates). If the mean across the 100 replications were close to the true values (the red dashed lines), estimation is unbiased. **Five Methods**: (1) **Full Data**: Ideal scenario where the substantive model is fitted to complete data (benchmark for the other methods). Four missing data handling methods applied to datasets with missing values: (2) **Bayes FIML**: Fitting the substantive model without missing data models but handling missingness with Bayesian FIML. (3) **Joint DV**: Joint modeling, simultaneously fitting the substantive and missing data models for two DVs. (4) **Joint TVcov**: Joint modeling, fitting the substantive and missing data models for two DVs and a time-varying covariate. (5) **Joint NoAR**: Joint modeling, fitting the substantive and missing data models for two DVs, but excluding AR terms from the missing data models. We displayed only the results for PA since the results for PA and EN were similar.

The **Joint TVcov** condition in Figure S1 is a variant of ’Joint DV’, with an additional missing data model for the time-varying covariate, alongside two DVs. The results closely resemble those of ’Joint DV’. While a slight improvement was observed in the C2 coefficient—where the true value of 0.1 was estimated at 0.11 due to addressing missing data in the time-varying covariate—this difference may not be substantial depending on research focuses. Thus, a pragmatic approach may be to include missing data models for the DVs as guided by theories, but incorporate simpler missing data handling methods on the time-varying covariates, such as partial FIML approach proposed by [27]. However, note that if missing data in time-varying covariates were largely MNAR, the gap between ’Joint TVcov’ and ’Joint DV’ might widen.

The **Joint NoAR** condition in Figure S1 is a simpler variant of ’Joint DV’. In this condition, substantive VAR model’s AR coefficients and baseline estimates showed slight bias, as shown in (a) and (d). The coefficient associated with the person-level covariate was also more biased compared ‘Joint DV’, which correctly included the AR term. This suggests that the lack of temporal structure in the missingness model limits its ability to capture correct substantial process, especially in ILD contexts where today’s compliance often depends on yesterday’s compliance.

Table S1. Simulated probit model results under the joint DV condition.

| Level | *Outcome* | Predictor | Parameter | True | Est. | rBias |
| --- | --- | --- | --- | --- | --- | --- |
| **Within-person** |  |  |  |  |  |  |
|  | ***Missing*_1,_*_i_*_,_*_t_*** |  |  |  |  |  |
|  |  | *Missing*_1,_*_i_*_,_*_t_*_− 1_ | *AR_y_*_1_ | 0.30 | 0.28 | -0.06 |
|  |  | *y*_1,_*_i_*_,_*_t_* | *ϕ_nmar_*_1,_*_y_*_1_ | 2.00 | 1.93 | -0.04 |
|  |  | *TVcov_i_*_,_*_t_* | *ϕ_mar_*_1,_*_y_*_1_ | 1.00 | 0.95 | -0.05 |
|  | ***Missing*_2,_*_i_*_,_*_t_*** |  |  |  |  |  |
|  |  | *Missing*_2,_*_i_*_,_*_t_*_− 1_ | *AR_y_*_2_ | 0.30 | 0.29 | -0.04 |
|  |  | *y*_2,_*_i_*_,_*_t_* | *ϕ_nmar_*_1,_*_y_*_1_ | -2.00 | -1.84 | -0.08 |
|  |  | *TVcov_i_*_,_*_t_* | *ϕ_mar_*_1,_*_y_*_2_ | 1.00 | 0.92 | -0.08 |
| **Between-person** |  |  |  |  |  |  |
|  | ***Intercept*_1,_*_i_*** |  |  |  |  |  |
|  |  | *yMean*_1,_*_i_* | *ϕ_nmar_*_2,_*_y_*_1_ | 2.00 | 2.51 | 0.26 |
|  |  | *TVcov_i_* | *ϕ_mar_*_2,_*_y_*_1_ | 1.00 | 1.53 | 0.54 |
|  |  | *x*2*_i_* | *ϕ_mar_*_3,_*_y_*_1_ | 1.00 | 1.60 | 0.60 |
|  | ***Intercept*_2,_*_i_*** |  |  |  |  |  |
|  |  | *yMean*_2,_*_i_* | *ϕ_nmar_*_2,_*_y_*_2_ | -2.00 | -2.26 | 0.13 |
|  |  | *TVcov_i_* | *ϕ_mar_*_2,_*_y_*_2_ | 1.00 | 1.48 | 0.48 |
|  |  | *x*_2_*_i_* | *ϕ_mar_*_3, 2_ | 1.00 | 1.54 | 0.55 |

*Note*. *Missing*_1,_*_i_*_,_*_t_*: a binary missingness indicator at time *t* for the *i*th person; True: true value of the parameter; Est.: estimated value of the parameter; rBias: relative bias (Est. – True)/True*100.
